# Proteomic Analysis of Plasmodesmata From *Populus* Cell Suspension Cultures in Relation With Callose Biosynthesis

**DOI:** 10.3389/fpls.2018.01681

**Published:** 2018-11-19

**Authors:** Felicia Leijon, Michael Melzer, Qi Zhou, Vaibhav Srivastava, Vincent Bulone

**Affiliations:** ^1^Division of Glycoscience, School of Engineering Sciences in Chemistry, Biotechnology and Health, Royal Institute of Technology, AlbaNova University Centre, Stockholm, Sweden; ^2^Leibniz Institute of Plant Genetics and Crop Plant Research, Gatersleben, Germany; ^3^ARC Centre of Excellence in Plant Cell Walls and School of Agriculture, Food and Wine, The University of Adelaide, Adelaide, SA, Australia

**Keywords:** *Populus*, plasmodesmata, callose synthase, callose, mass spectrometry, spectral counting

## Abstract

Plasmodesmata are channels that link adjacent cells in plant tissues through which molecular exchanges take place. They are involved in multiple processes vital to plant cells, such as responses to hormonal signaling or environmental challenges including osmotic stress, wounding and pathogen attack. Despite the importance of plasmodesmata, their proteome is not well-defined. Here, we have isolated fractions enriched in plasmodesmata from cell suspension cultures of *Populus trichocarpa* and identified 201 proteins that are enriched in these fractions, thereby providing further insight on the multiple functions of plasmodesmata. Proteomics analysis revealed an enrichment of proteins specifically involved in responses to stress, transport, metabolism and signal transduction. Consistent with the role of callose deposition and turnover in the closure and aperture of the plasmodesmata and our proteomic analysis, we demonstrate the enrichment of callose synthase activity in the plasmodesmata represented by several gene products. A new form of calcium-independent callose synthase activity was detected, in addition to the typical calcium-dependent enzyme activity, suggesting a role of calcium in the regulation of plasmodesmata through two forms of callose synthase activities. Our report provides the first proteomic investigation of the plasmodesmata from a tree species and the direct biochemical evidence for the occurrence of several forms of active callose synthases in these structures. Data are available via ProteomeXchange with identifier PXD010692.

## Introduction

Plasmodesmata are PM-lined pores that connect the intracellular compartments of adjacent plant cells through cytoplasmic sleeves, thereby permitting symplastic transport (Roberts and Oparka, 2003). The movement of molecules such as nutrients, transcription factors, and nucleic acids through the PD is determined by the size exclusion limit of the channel, and the regulation of its opening and closure (de Storme and Geelen, 2014). The PD contains a so-called desmotubule, comprised of a small section of ER membrane. Various cytoskeletal elements and multiple proteins are also localized at the PD, and some of these components might be involved in the control of intercellular molecular flow (Ding et al., 1996; Radford and White, 1998; Baluška et al., 2001; Wu et al., 2018). Trafficking is regulated in response to hormonal signaling or environmental challenges such as osmotic stress, chilling, wounding, pathogen attack, and the presence of toxic substances (Schulz, 1995; Sivaguru et al., 2000; Bilska and Sowinski, 2010; Cui and Lee, 2016). Recently, it has been shown that the growth hormone abscisic acid is involved in establishing dormancy in *Populus* by blocking intercellular communication through the PD (Tylewicz et al., 2018), suggesting a vital role in the fundamental biology of the tree. The growth hormone gibberellic acid is involved in dormancy breakage and the subsequent degradation of callose plugs in *Populus* (Rinne et al., 2011), and auxin has also been implicated in the regulation of callose biosynthesis at the PD (Han et al., 2014). In addition, PD are involved in the transmission of viral particles between cells (Niehl and Heinlein, 2011).

Reduced cell-to-cell movement through the PD can typically be attributed to the deposition of callose, a linear 1,3-β-glucan, in the cell walls surrounding the pore structure (Sivaguru et al., 2000; Rinne et al., 2001; Vatén et al., 2011), although non-callose-dependent closure may also occur (Sager and Lee, 2014). The hydrolytic removal of callose and subsequent opening of the PD is performed by 1,3-β-glucanases (Iglesias and Meins, 2000; Levy et al., 2007; Benitez-Alfonso et al., 2013). Callose has been shown to be associated with many stages of plant development (Rinne et al., 2001; Ruan et al., 2004; Wilson et al., 2006; Chen et al., 2009). It is also deposited as a response to both biotic and abiotic stress (Sivaguru et al., 2000; Jacobs et al., 2003). The enzymes responsible for the formation of callose are members of the callose synthase family, commonly referred to as ‘glucan synthase-like’ (GSL) proteins. These are classified as processive glycosyltransferases (GT) belonging to CAZy family GT48 (Coutinho et al., 2003; Lombard et al., 2014) and considered to be part of a larger complex (Verma and Hong, 2001). Although GSLs have never been purified to homogeneity for detailed biochemical characterization of their activity, indirect evidence of callose synthase activity derives from activity assays performed on semi-purified membrane fractions (Li et al., 2003; Brownfield et al., 2007) and mutational studies (Thiele et al., 2009; Guseman et al., 2010; Vatén et al., 2011).

While the evidence for callose deposition at PD is substantial, the precise composition of the enzyme complexes involved in this process has so far been elusive. In *Arabidopsis* the GSL family has 12 members (Hong et al., 2001), which may be specific to certain tissues and involved in specific processes (Ellinger and Voigt, 2014). Among these, five callose synthases (GSL4, 6–8, and 12) have been directly implicated in the formation of callose at the PD. It has been shown that loss-of-function GSL8 mutants have reduced callose accumulation at the PD, and an increase in cell-to-cell transport (Guseman et al., 2010). Likewise, together with defects in vascular tissue, an increased callose deposition and reduced PD connectivity were found in gain-of-function mutants of *At*GSL12 (Vatén et al., 2011). GSL7 is essential for normal phloem transport in *Arabidopsis* (Barratt et al., 2011). It was also shown that GSL7 mutants present a reduction in the amount of pores in their phloem sieve plates due to reduced callose deposition in precursor PD (Xie et al., 2011). In addition, GSL4 and GSL6 are respectively involved in PD callose deposition in response to ROS and SA signaling (Cui and Lee, 2016).

Attempts to locate individual proteins to the PD has identified enzymes, structural proteins, and regulatory proteins involved in various PD functions (Sagi et al., 2005; Levy et al., 2007; Thomas et al., 2008; Raffaele et al., 2009; Fernandez-Calvino et al., 2011; Vatén et al., 2011; Ham et al., 2012). However, while localization experiments can give definitive proof of a protein’s presence at the PD, such investigations do not give an overall view of the PD proteome, and nor are they useful for uncovering new proteins. MS based proteomics approaches are of greater utility in this area. The most extensive list of PD derived proteins so far was published by Fernandez-Calvino et al. (2011), and described the qualitative proteome of *Arabidopsis* PD. More recently, through quantitative proteomics, Grison et al. (2015a) showed that PD-associated proteins are highly enriched in the PD fraction compared with the bulk PM in *Arabidopsis*.

In our previous work on *Populus* cell membrane proteins (Srivastava et al., 2013), we proposed that a subset of proteins found in DRMs might derive from the PD, due to the enrichment of callose synthases and other typical PD associated proteins in the DRM samples. Here, we have directly investigated the proteome of the PEF from poplar cell suspension cultures and quantitatively compared its protein composition with other membrane fractions. We build on the previously published literature on the PD proteome by using semiquantitative methods and, for the first time, demonstrate *in vitro* callose synthase activity from *Populus* PD proteins. Our new data show that part of the callose synthase activity in the PEF is cation-independent, suggesting a novel mode of action. This is the first direct biochemical evidence of the existence of two forms of callose synthase activity at the PD, which supports our proteomic data and, to our knowledge, the first proteomic investigation of the PD performed on a tree species.

## Materials and Methods

### Cell Fractionation and Preparation of Plasmodesmata

Cell suspension cultures of *Populus trichocarpa* were generated as previously described (Ohlsson et al., 2006). Cultures were maintained under rotational agitation (125 rpm) at 24°C and a 12 h light/12 h dark regime. Cells were subcultured on a weekly basis in Murashige and Skoog medium supplemented with sucrose (3%), 2, 4-dichlorophenoxyacetic acid (1 mg/l), and kinetin (0.02 mg/l) (Colombani et al., 2004). They were harvested during the logarithmic growth phase 8 days after inoculation and washed on Miracloth (Calbiochem, Germany) by vacuum filtration with ice-cold 100 mM Tris-HCl buffer (pH 8). All subsequent steps were performed at 4°C.

**FIGURE 1:**
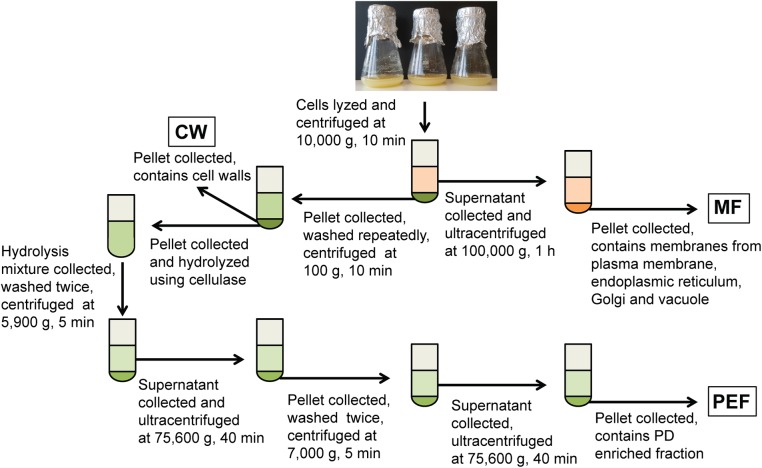
Overview of the experimental set-up for the fractionation of membranes from cell suspension cultures. Fractions collected for callose synthase assays and/or MS analysis are shown in boxes. MF, microsomal fraction; CW, cell wall fraction; PEF, plasmodesmata enriched fraction.

Cell walls were prepared as in Fernandez-Calvino et al. (2011) and Grison et al. (2015b), with the following modifications. Briefly, cells were resuspended in lysis buffer consisting of 100 mM Tris-HCl pH 8 (1 ml/g fresh cells) supplemented with 100 mM KCl, 10% glycerol, 10 mM EDTA, 0.45 M mannitol and protease inhibitors (‘complete’ mini tablets, Roche), and disrupted at 80 MPa using a French press (SLM-Amicon Instruments, Thermo Spectronic). The homogenate was centrifuged at 10,000 × *g* for 10 min and the resulting supernatant and cell wall pellet were collected separately (Figure 1). The supernatant was filtered through Miracloth and centrifuged at 100,000 × *g* for 1 h to sediment the microsomal membranes. These were resuspended in 10 mM MOPS buffer pH 7.5 supplemented with protease inhibitors as indicated above. Protein concentration in the MF was determined using the Bradford dye-binding assay (Bio-Rad) with bovine serum albumin as a standard. The cell wall pellet was resuspended in lysis buffer (5 ml/g of starting fresh cells) and the suspension was centrifuged at 500 × *g* for 10 min. The resulting pellet corresponding to enriched cell walls (CW) was resuspended and washed repeatedly by centrifugation at 100 × *g* for 5 min in 10 mM Tris-HCl pH 8 containing 100 mM NaCl, 10% glycerol and 10 mM EDTA. The final pellet was resuspended in 10 mM MES pH 5.5 containing 0.24 M mannitol and protease inhibitors.

The PEF was prepared from the CW sample using a cellulase treatment (cellulase R10 from *Trichoderma viride*; Karlan, United States) and subsequent washing and centrifugation steps as described in Fernandez-Calvino et al. (2011) and summarized in Figure 1. For callose synthase assays, the CW, MF, and PEF pellets were resuspended in 100 mM Tris-HCl pH 7 containing 10% glycerol and protease inhibitors and the assays were carried out as described below.

### Proteomic Analysis

Semi-quantitative proteomic analysis was performed on the MF and PEF fractions after SDS-PAGE separation of the proteins in each sample using 12% Tris-Glycine Mini Protean TGX gels from BioRad. After staining with Coomassie Blue (ThermoScientific, United States), each lane of the gel was cut into 25 bands and the proteins were subjected to in-gel digestion with trypsin as previously described (Srivastava et al., 2013). Peptide analysis was performed with reverse-phase LC–electrospray ionization–MS/MS using a nanoACQUITY Ultra Performance Liquid Chromatography system coupled to a Q-TOF mass spectrometer (Xevo Q-TOF, Waters, Milford, MA, United States). The in-gel tryptic peptides were resuspended in 0.1% TFA and loaded on a C18 trap column (Symmetry 180 μm × 20 mm, 5 μm; Waters, Milford, MA, United States) that was then washed with 0.1% (v/v) formic acid at 8 μl/min for 7 min. The samples eluted from the trap column were separated on a C18 analytical column (75 μm × 150 mm, 1.7 μm; Waters, Milford, MA, United States) at 250 nl/min using 0.1% formic acid as solvent A and 0.1% formic acid in acetonitrile as solvent B in a stepwise gradient: 0–10% B (0–5 min), 10–30% B (5–80 min), 30–40% B (80–92 min), 40–90% B (92–95 min), 90% B (95–100 min), and 90–0.1% B (100–102 min). The eluting peptides were sprayed in the mass spectrometer (capillary and cone voltages set to 2.3 kV and 45 V, respectively), and MS/MS spectra were acquired using automated data-directed switching between the MS and MS/MS modes using the instrument software (MassLynx V4.0 SP4). The five most abundant signals of a survey scan (400–1600 m/z range, 1s scan time) were selected by charge state, and collision energy was applied accordingly for sequential MS/MS fragmentation scanning (100–1800 m/z range, 1s scan time).

The APP (Malm et al., 2014), which utilizes multiple search engines, was used to analyze the MS data using the Populus protein database (Populus version 3.0; 73,013 entries) with the following settings: trypsin specific hydrolysis with two missed cleavages allowed; peptide tolerance of 100 ppm; fragment tolerance of 0.5 Da; oxidized Met as variable modification and ethanolated Cys as fixed modifications. A concatenated target-decoy database-search strategy showed false positive rates of less than 1% in all searches.

For each protein, peptide sequences were exported with a protein and peptide probability cutoff of 0.95. Peptides matching two or more proteins (shared peptides) were excluded from the analysis as were proteins with no unique peptides (i.e., identified by shared peptides only). A protein was considered identified if it contained at least one unique peptide. For each BR, the final protein list obtained after the ProteinProphet step was submitted to an in-house plugin for spectral counting. Unique peptide sequences were filtered out for each protein, using a protein and peptide probability cutoff of 0.95. For these peptides, all matching spectra above 0.5 probabilities were indexed for spectral counting. Spectral counts were normalized to the total number of spectra counted, which gave a NSAF for each protein. The NSAF ratio was calculated only for proteins identified in both MF and PEF samples. The protein list obtained from all three BRs was further filtered in order to identify proteins most enriched in the PEF. A final list of proteins was assembled. It consisted of proteins that were detected in at least two of the three BRs with two or more unique peptides per BR, and proteins detected in all three BRs with one or more unique peptide per BR. Proteins from this selected group were considered PEF enriched when present in PEF only or when their NSAF ratio (PEF/MF) was four or higher in at least two independent BRs. The MS proteomics data have been deposited to the ProteomeXchange Consortium via the PRIDE (Vizcaíno et al., 2016) partner repository with the dataset identifier PXD010692.

### *In silico* Prediction of Post-translational Modifications and Protein Topology

Glycosylphosphatidylinositol anchors, myristoylation and palmitoylation sites were predicted from the sequences of all identified proteins using PredGPI predictor (Pierleoni et al., 2008), Plant-Specific Myristoylation Predictor (Podell and Gribskov, 2004) and CSS-Palm 3.0 (Ren et al., 2008), respectively. Signal peptides were identified using Signal P version 4.1 (Petersen et al., 2011) and TMDs were predicted using HMMTOP (Tusnády and Simon, 2001). Theoretical molecular masses and isoelectric points were calculated using the Compute pI/Mw tool (ExPASy^1^). Gene ontology information was retrieved using The Arabidopsis Information Resource (TAIR^2^) and Blast2Go (Conesa et al., 2005). Protein kinases were analyzed and classified using iTAK (Zheng et al., 2016).

### Transmission Electron Microscopy (TEM)

For ultrastructural analysis, conventional, and microwave assisted fixation, substitution, and resin embedding of isolated cells was performed as described in Supplementary Table S1. Ultrathin sections of approximately 70 nm were cut with a diamond knife, transferred onto TEM grids and contrasted in a LEICA EM STAIN (Leica Microsystems, Vienna, Austria) with uranyl acetate and Reynolds’ lead citrate. Ultrastructural analysis was performed using a Tecnai Sphera G2 TEM (FEI, Eindhoven, Netherlands) operated at 120 kV.

### Callose Synthase Assay

For callose synthase assays, the CW, MF, PEF fractions (Figure 1) were incubated for 16 h at 24°C in 100 mM Tris-HCl buffer pH 7, containing 8 mM CaCl_2_, 0.8 mM UDP-glucose and 0.76 μM UDP-[U-^14^C]glucose (263 mCi/mmol; Perkin Elmer, Boston, MA, United States) (Brown et al., 2012). To test the effect of calcium on callose synthase activity, assays were repeated in the same reaction mixtures as above, except for the addition of 10 mM EDTA and the omission of CaCl_2_. All assays were performed in triplicates on at least three BRs for each sample type. The insoluble product formed in the callose synthase reaction mixtures was characterized by enzymatic hydrolysis using the specific endo-1,3-β-glucanase from *Trichoderma* sp. (50 U/ml) (Megazyme, Ireland). For this purpose, the reaction mixtures were centrifuged at 21,000 × *g* for 15 min and the resulting pellets containing insoluble radioactive polysaccharides were resuspended in 50 mM sodium acetate buffer pH 4.5, and subjected to enzymatic hydrolysis for 5 h at 40°C in the presence of 0.026 enzyme unit. In these conditions, one unit of enzyme releases 1 μmol of glucose from carboxymethylated curdlan. Negative controls were prepared by replacing the hydrolytic enzyme with acetate buffer.

## Results and Discussion

### Analysis of the Plasmodesmata Proteome

**FIGURE 2:**
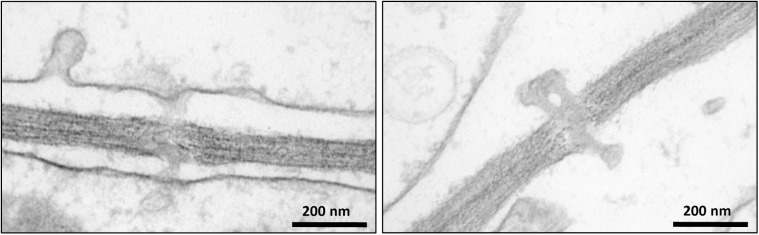
Transmission electron microscopy images showing the occurrence of plasmodesmata in poplar cells.

Intercellular transport through PD plays a crucial role in the development and growth of plants (Zavaliev et al., 2010; Vatén et al., 2011; Benitez-Alfonso et al., 2013). Despite the fundamental importance of PD, a limited number of studies have investigated their protein composition (Faulkner et al., 2005; Fernandez-Calvino et al., 2011). One of the bottlenecks for reliable proteomic analysis of PD is their purification from the carbohydrate-rich cell wall matrix. Attempts have been made to identify PD associated proteins through immunolocalization of known proteins (Blackman and Overall, 1998; Radford and White, 1998) and mass-spectrometry proteomic strategies (Faulkner et al., 2005; Fernandez-Calvino et al., 2011). In addition, mutant and high-throughput screens of random cDNAs have been performed to search for PD-associated proteins (Kim et al., 2002; Escobar et al., 2003). Here we have used a quantitative proteomic approach combined with biochemical assays to further expand our knowledge of the composition and function of PD (Supplementary Figure S1). Cell suspension cultures were used as a convenient system for our investigations to overcome the challenging extraction of membrane structures from woody tissues. Bayer et al. (2004) have shown that *Arabidopsis* cells in suspension cultures predominantly form unbranched PD, probably corresponding to primary PD. Since suspension cultures were used in our study to isolate PD, it can be hypothesized that similar types of PD structures also occur in poplar cell cultures. The occurrence of PD in *Populus* cell suspension cultures was confirmed by electron microscopy imaging (Figure 2). The isolated structures were found in high abundance in the samples prepared from cell walls of *Populus trichocarpa* cells, with a typical morphology of intercellular channels spanning across cell walls of adjacent cells. The protocol used to isolate PD was similar to that described in Fernandez-Calvino et al. (2011) for the preparation of PD from *Arabidopsis* cell suspension cultures, which makes our study directly comparable to this earlier work. The list of PD proteins obtained from the *Arabidopsis* samples is the most comprehensive available to date (Fernandez-Calvino et al., 2011). However, out of the 1341 proteins identified in these samples approximately 35% were reported to be cytoplasmic contaminants not related to PD function (Fernandez-Calvino et al., 2011).

A total of 1113 unique proteins were identified from all three BR of the *Populus* PEF samples (Supplementary Table S2). Comparison of the list of proteins identified in each BR revealed that 355 were common to all replicates. However, each replicate also contained proteins not identified in the other two replicates (Supplementary Table S2). Approximately, 62% of these were predicted to be located at the PM, cell wall or present extracellularly (Supplementary Table S2). Around 7% of the proteins were ribosomal or ER related. As also shown for the *Arabidopsis* PD (Fernandez-Calvino et al., 2011), about 27% of the proteins identified in our PEF samples most likely originated from other cell compartments that co-purified with the PEF, i.e., the nucleus, Golgi apparatus, cytosol, plastids, and mitochondria (Supplementary Table S2). To select proteins that are specific to PD we further applied strict filtering criteria (see section “Materials and Methods”). This led to the identification of a total of 201 proteins enriched in PD (Supplementary Table S2). This selected group contains numerous proteins previously localized to PD such as members of the PDLPs, tetraspanins, RGPs, remorin, calreticulin, and callose synthase (GSLs), indicating a true enrichment of PD in our PEF samples.

### Functional Classification, Localization, and Biochemical Properties of PD Enriched Proteins

**FIGURE 3:**
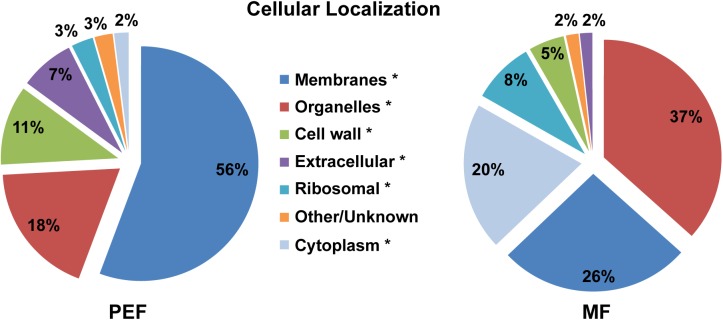
Predicted cellular localization of proteins enriched in the PEF and the MF. The asterisks mark the localization categories of proteins that present significantly different levels of enrichment between the PEF and MF samples (*p* < 0.05; Chi-square test).

**FIGURE 4:**
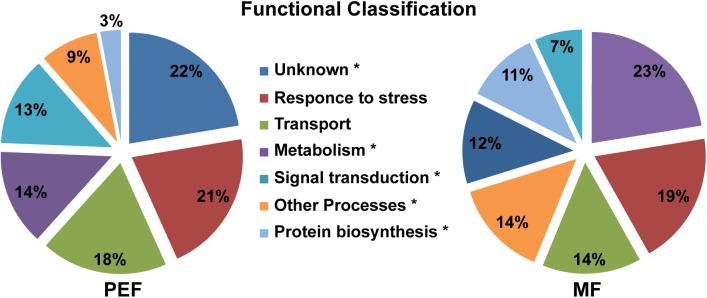
Predicted functional classification of proteins enriched in the PEF and the MF. The asterisks mark the functional categories of proteins that present significantly different levels of enrichment between the PEF and MF samples (*p* < 0.05; Chi-square test).

The predicted cellular localization and functional classification of the proteins enriched in the *Populus* MF and PEF samples are presented in Figures 3, 4, respectively. Fifty-six percent of the PD enriched proteins were classified as membrane-bound (Figure 3). Smaller groups representing organelles (18%, 1/3 ER derived), cell wall (11%), and extracellular proteins (7%) were also present in the PEF samples. As the PD are integral structures of the cell wall, it is not surprising that a subset of enriched proteins is classified as cell wall located or extracellular. Despite the use of stringent filtering criteria, a small percentage of proteins related to intracellular organelles and membrane compartments (mitochondrial, plastid, nucleus, chloroplast, ER) were detected in the PEF samples (Figure 3). With the exception of proteins related to ER, which may have a desmotubular origin, these proteins most likely arose from contaminations. As expected, the percentage of organelle localized (37%) and cytoplasmic (20%) proteins were significantly higher in the MF samples (Figure 3).

The list of proteins enriched in both MF and PEF samples was compared with the information available in the TAIR database. The closest homologs of a large fraction (43%) of the *Populus* proteins enriched in the PEF sample were annotated as PD-localized in *Arabidopsis* (Supplementary Table S2). This observation is in good agreement with the previous studies which further confirms the reliability of the protocol used to prepare our PD fraction. Similarly, 18% of the *Populus* proteins enriched in the MF sample were also reported as PD located in *Arabidopsis* (Supplementary Table S2). This relatively high proportion may be due to the dynamic nature of some PD proteins that are also involved in other signaling and transport pathways and that transit to or from the PD. Organelle enrichment was also evaluated using the Multiple Marker Abundance Profiling (MMAP) tool from the SUBA Toolbox (Hooper et al., 2017^3^). The relative protein abundance map showed that 98% of all PEF proteins were classified as PM localized and extracellular (Supplementary Figure S2). These observations not only confirm the efficiency of the protocol used to isolate PD, but also show that the filtering criteria we used are appropriate for the selection of PD proteins.

The proteins enriched in the PEF samples belong to several categories, namely response to stress and defense (21%), transport (18%), metabolism (14%) and signal transduction (13%), while 22% have an unknown function (Figure 4 and Supplementary Table S2). The MF samples contained proteins related to the same categories, but in different proportions. Proteins from the metabolism (23%) and response to stress and defense (19%) categories were in highest proportion followed by transport proteins (14%). Compared to PEF, a significantly smaller proportion of MF proteins (12%) have an unknown function (Figure 4).

**FIGURE 5:**
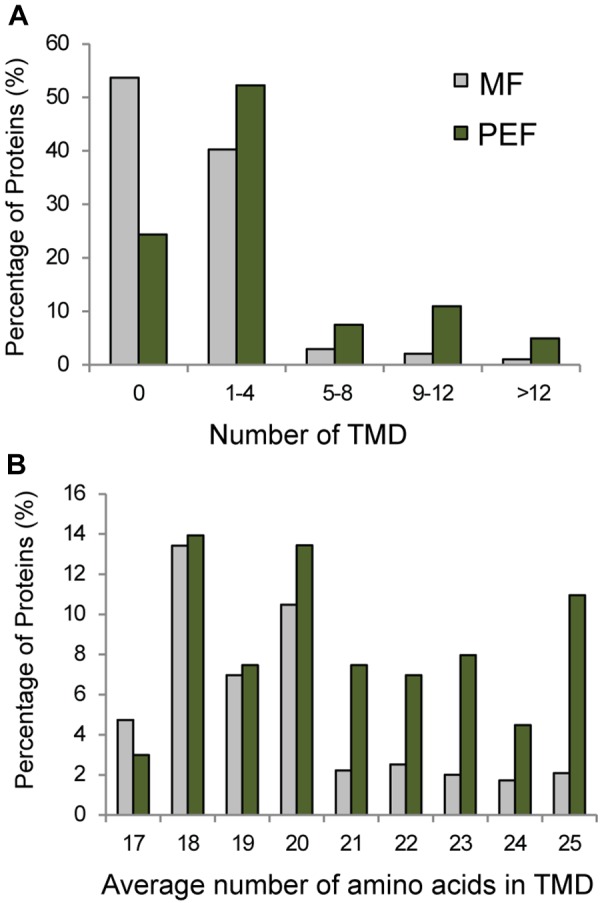
Grouping of PEF and microsomal (MF) proteins based on their number of TMDs **(A)** and average number of amino acids in TMD **(B)**.

Seventy six percent of the proteins found in PEF are predicted to contain one or more TMDs (Figure 5A). Interestingly, these proteins are characterized on average by a higher number of predicted TMDs compared with the proteins enriched in the MF sample (Figure 5B). In addition, the PD enriched proteins tend to contain a higher number of amino acids per TMD (Figure 5B). Indeed, a TMD length of 21 amino acids or more was found in a significantly higher proportion of proteins from the PEF (38%) sample compared to MF (11%) (Figure 5B). Membrane domains composed of phospholipids with long chain fatty acids are predicted to be thicker than non-raft bilayers (Gandhavadi et al., 2002) and able to accommodate proteins with longer TMDs (Bretscher and Munro, 1993; McIntosh et al., 2003). Earlier data on PD membrane composition also showed a higher proportion of sterols and sphingolipids with very long chain saturated fatty acids compared to PM (Grison et al., 2015a). These observations are in agreement with a higher number of amino acids in the predicted TMDs of proteins from the PEF sample. It is well-known that post-translational modifications such as acylation and glypiation are involved in membrane association of proteins. Among the 201 PEF-enriched proteins, 104 are predicted to be palmitoylated, 3 myristoylated and 9 GPI anchored (Supplementary Table S2). In addition, a greater proportion of PEF proteins had a higher mass range (51–125 kDa) and isoelectric point (≥8) (Supplementary Figure S3). The dominance of more basic proteins is most likely due to a high occurrence of integral membrane proteins in the PEF sample (Schwartz et al., 2001).

### Analysis of Specific Protein Classes in Plasmodesmata

The PEF enriched proteins identified in our study were predicted to be involved in diverse cellular processes (Figure 4 and Supplementary Table S2). In the following subsections we discuss the most prominent proteins and protein classes in relation to their potential function at the PD.

### Proteins Related to Callose Metabolism

Several proteins involved in callose biosynthesis and degradation were found to be enriched in the PEF sample (Supplementary Table S2). Accumulation and turnover of callose at the PD is mainly determined by three classes of proteins, namely GSL proteins that synthesize callose, 1-3-β-D-glucanases that degrade callose and some other proteins that stabilize callose (Ueki and Citovsky, 2014). The *Populus trichocarpa* genome contains at least 13 putative GSL genes (Tuskan et al., 2006). The numbering used here for the identified proteins is based on sequence similarity with the *Arabidopsis* callose synthases (Supplementary Figure S4). Three callose synthases (GSL5, GSL8, and GSL10) were identified in both MF and PEF samples. However, only GSL5 and GSL10 were found to be highly enriched in PEF (Supplementary Table S2). GSL5 has been previously linked to callose deposition as a stress response in connection to wounding and fungal penetration (Jacobs et al., 2003; Nishimura et al., 2003) and is essential for pollen maturation (Enns et al., 2005). Both GSL8 and GSL10 are also involved in pollen development (Töller et al., 2008). GSL8 has been further associated with callose synthesis at the PD (Guseman et al., 2010; Han et al., 2014) and mediates basal PD permeability in *Arabidopsis* (Cui and Lee, 2016). It is also involved in callose deposition at the cell plate (Chen et al., 2009; Thiele et al., 2009). The presence of GSL8 in both PEF and MF samples without any clear enrichment in either fraction, could be explained by a high level of cell division taking place in the cell cultures as the cells were harvested in logarithmic growth phase. The callose synthase GSL12, which regulates symplastic trafficking via PD during root development (Vatén et al., 2011), was found only in the PEF samples (Supplementary Table S2). Grison et al. (2015a) also found GSL12 to be enriched in PD samples compared to PM purified from *Arabidopsis* cell suspension cultures. In our previous work, several forms of callose synthases were reported to be enriched in DRM isolated from *Populus* cell cultures and it was hypothesized that such domains also occur in PD (Srivastava et al., 2013).

The PEF sample contains four putative glucanases from the GHs 17 family that are predicted to hydrolyse 1,3-β-glucans (Supplementary Table S2). These enzymes play an important role in the turnover of callose at the PD and the conductivity of the PD is detrimentally affected by the loss of such hydrolases (Iglesias and Meins, 2000; Levy et al., 2007; Zavaliev et al., 2013). The presence of several glucanases suggests that several callose degrading regulatory pathways are involved in the dynamics of PD structure, but this needs to be further investigated.

Several “purple” acid phosphatases (PAP) were also enriched in PEF (Supplementary Table S2). Overexpression of PAPs has been linked to the activation of callose synthases and was hypothesized to be involved in the regulation of the enzyme through phosphorylation/dephosphorylation (Kaida et al., 2009). Other proteins previously proposed to be part of the callose synthase complex, such as the ROP, UGT, phragmoplastin, SUSY, and annexin were not identified in the PEF samples (Andrawis et al., 1993; Amor et al., 1995; Verma and Hong, 2001). This indicates that either the concentration of these proteins was too low in the PEF sample or they may not be associated with the PD callose synthase complex. It can also be hypothesized that callose synthase complexes located at the PD have a different composition than those found in other parts of the cell.

### Callose Synthase Activity in Plasmodesmata

**FIGURE 6:**
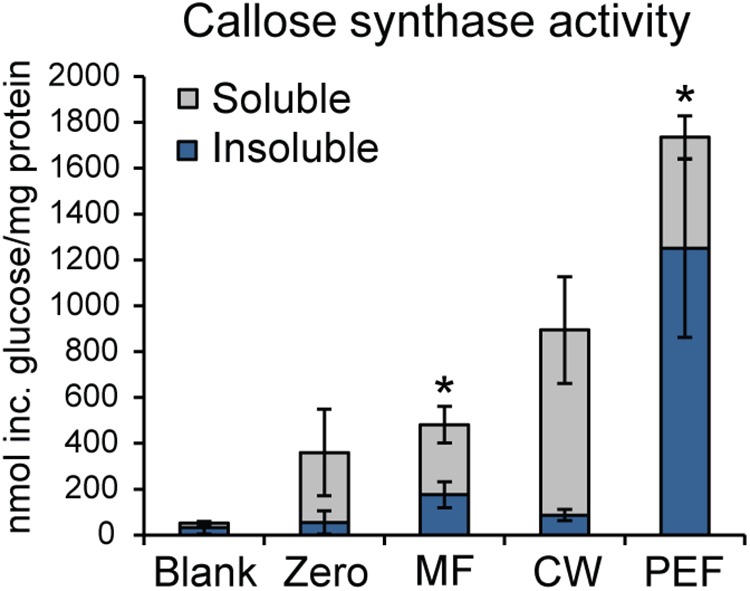
Enrichment of callose synthase activity in fractions derived from *Populus trichocarpa.* Both insoluble and soluble product formation was assayed. ‘Blank’ samples had no protein added and reactions corresponding to the ‘Zero’ samples were stopped immediately with ethanol. Activity is presented as nmol of glucose incorporated per mg total protein used for the assay. Reactions were run at room temperature for 16 h. Values are based on five biological replicates and three technical replicates for each BR. MF, microsomal fraction; CW, cell wall fraction; PEF, plasmodesmata enriched fraction. Significant differences between the MF and PEF samples (columns marked by asterisks) were found for both soluble and insoluble products (*p* < 0.001 for both the insoluble and soluble products; Student’s *t*-test). Bars indicate standard deviations.

As callose is considered a marker for PD, the presence of callose synthase activity was also used to verify the enrichment of PD in the PEF sample. Callose synthase activity was assayed in the MF, the CW, and the PEF. Callose synthase activity was clearly enriched in the PEF sample compared to MF (Figure 6), hence confirming the enrichment of PD derived membranes. When Ca^2+^ was present in the reaction mixture, the principal product formed *in vitro* by the PEF membranes was an insoluble glucan, while a higher proportion of soluble product was synthesized by the MF samples (Figure 6). The formation of 1,3-β-glucan by the MF and PEF membranes was confirmed using a specific endo-1,3-β-glucanase, which completely degraded the radioactive *in vitro* product after 5 h incubation (Supplementary Figure S5a).

**FIGURE 7:**
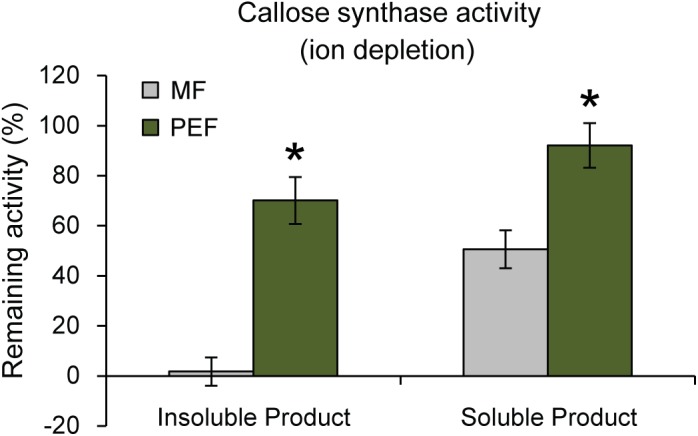
Remaining callose synthase activity under ion depleted conditions (10 mM EDTA in the reaction mixture and no calcium added). Activity is presented as % remaining activity, which is the activity under ion depleted conditions with respect to the activity under calcium-dependent condition. Values are based on four BRs and three technical replicates for each BR (*p* < 0.001 for both the insoluble and soluble products; Student’s *t*-test). Bars indicate standard deviations.

Interestingly, it has been shown that PD closure is regulated by calcium levels in the cytoplasm (Tucker and Boss, 1996; Holdaway-Clarke et al., 2000). Since a majority of reported callose synthase activities are Ca^2+^ dependent (Köhle et al., 1985; Schlüpmann et al., 1993; Colombani et al., 2004) and callose deposition at PD leads to lower conductivity (Sivaguru et al., 2000; Rinne et al., 2005; Levy et al., 2007; Guseman et al., 2010; Vatén et al., 2011), a possible connection between Ca^2+^-regulated PD closure and callose deposition was investigated. If callose synthase activity at the PD is Ca^2+^ dependent, calcium levels may be one of the regulatory ways that controls PD aperture by controlling callose deposition. Callose synthase activity assays were therefore performed in the absence of calcium in the reaction mixture. This showed that the formation of insoluble and soluble glucans by the PEF sample decreased by 30% and 8% in the absence of calcium, respectively, but that it was not abolished (Figure 7). In addition, activity in the MF sample showed the expected cation dependence and virtually no insoluble product was detected in the absence of calcium while the amount of soluble product decreased by approximately 49% (Figure 7). The existence of a Ca^2+^-independent callose synthase activity has previously been reported in and associated to pollen tube development (Schlüpmann et al., 1993), whereas all other types of callose synthases are known to be calcium dependent. Product characterization performed on the PEF product formed in the absence of calcium resulted in full degradation of the glucan synthesized *in vitro* (Supplementary Figure S5b). This data confirmed that calcium depletion had no effect on the type of polysaccharide synthesized (Supplementary Figure S5b).

Altogether, these data indicate that the PEF and MF samples contain both calcium-dependent and calcium-independent callose synthase activities, with a higher proportion of the calcium-independent form in the PD. It can be proposed that several forms of the enzyme co-exist at the PD and that these are involved in the regulation of PD activity. Additional experiments on isolated forms of PD callose synthase activities would be needed to confirm this hypothesis. It remains nonetheless that our data reveal the existence of a second form of calcium-independent callose synthase activity similar to the previously reported developmental pollen tube enzyme (Schlüpmann et al., 1993).

### Cell Wall Associated Proteins

Cell walls surrounding the PD have a distinct composition with pectins as major components, and, transiently, callose (Faulkner et al., 2008; Knox and Benitez-Alfonso, 2014; Amsbury et al., 2018). Many proteins involved in cell wall remodeling and growth were found in the PD samples (Supplementary Table S2). As the PD structure is dynamic and changes depending on the developmental stages and cell/tissue type (Burch-Smith et al., 2011) it is possible that these proteins are involved in ultrastructural modifications of the PD. Several RGPs were enriched in the PEF (Supplementary Table S2). These are UDP-arabinose mutases (Konishi et al., 2007; Rautengarten et al., 2011) that are transported to the PD via Golgi vesicles (Sagi et al., 2005). Down-regulating RGP expression leads to increased PD permeability (Burch-Smith and Zambryski, 2012), while constitutive expression reduces permeability as a result of RGP or callose accumulation at the PD (Zavaliev et al., 2010). Several GHs of the glucosidase and mannosidase types were also enriched in the PEF and possibly involved in carbohydrate turnover or glycoprotein modification. Another group of enriched proteins involved in cell wall development and assembly are the LRXs (Baumberger et al., 2001; Draeger et al., 2015). Additional PEF proteins involved in cell wall modifications are expansin-like protein, a COBRA-like protein, a monocopper oxidase and a FAD-binding Berberine family protein, all of which have previously been linked to PD (Fernandez-Calvino et al., 2011).

Pectine methylesterases were identified in the PD sample without a significant level of enrichment. These have been previously identified in a PD proteome (Fernandez-Calvino et al., 2011) and found in the cell wall around PD (Morvan et al., 1998; Chen et al., 2000) where they are hypothesized to participate in cell wall remodeling in the vicinity of the PD structure.

### Proteins Related to Stress Responses

Response to stress, the second largest functional category of the PD enriched proteins, is represented by several large groups (Supplementary Table S2). Two ERD stress proteins (Kiyosue et al., 1994) were found in the PEF (Supplementary Table S2). Similarly, several representatives from the LEAs and NDR1/HIN1-like proteins were also present (Supplementary Table S2). These proteins have been shown to play an important role during viral and bacterial infection (Dörmann et al., 2000; Varet et al., 2002; Zheng et al., 2004). Germin-like proteins whose overexpression is related with increased transport through PD (Ham et al., 2012) were also enriched in the PEF samples (Supplementary Table S2).

Peroxidases were also identified in the PEF samples. Ehlers and van Bel (2010) showed the presence of peroxidases near the PD through immunolocalization and suggested that they contribute to PD structural alterations by cell wall loosening through the production of hydroxyl radicals. Fernandez-Calvino et al. (2011) also identified several class III peroxidases in the *Arabidopsis* PD proteome.

Calreticulin was also enriched in the PEF samples. This protein is predominant in the ER (Denecke et al., 1995) and is likely associated with the desmotubule (Baluška et al., 2001; Bayer et al., 2004). It is mainly involved in Ca^2+^ sequestration and signaling and in chaperone activity (Michalak et al., 1998; Persson et al., 2001). Calreticulin interacts with a tobacco mosaic virus movement protein and its targeting to the PD is impaired under elevated calreticulin levels (Chen et al., 2005). Its upregulation is also correlated to stress response with callose deposition at the PD resulting in reduced cell-to-cell movement (Sivaguru et al., 2000). Consistent with this observation, it was hypothesized that PD localized calreticulin accumulates Ca^2+^, which results in activation of callose synthases and the subsequent callose dependent closure of PD (de Storme and Geelen, 2014). However, other authors speculated that calreticulin activity is unrelated to callose deposition and that it is involved in sequestering Ca^2+^ as part of a negative feedback loop regulating the Ca^2+^ dependent closure of PD (Sager and Lee, 2014).

### Intracellular Trafficking and Membrane Interacting Proteins

As PD is a membrane rich structure where PM and desmotubule membranes lie in close proximity to each other, it is probable that structural and signal transduction proteins involved in maintaining and regulating these membrane contact sites are present at the PD (Tilsner et al., 2016). Here, we detected proteins associated with the SNARE complex involved in fusion between vesicle and acceptor membranes. Several q-SNAREs (syntaxins), r-SNAREs (VAMPS) and SNAP proteins were enriched in the PD (Supplementary Table S2). The *Arabidopsis* homologs of several of these proteins were found to be PM or ER located (Uemura et al., 2004). Among these a q-SNARE (SYP121/PEN1) was previously shown to co-immunoprecipitate with the PDLP1 protein from *Arabidopsis* membranes (Caillaud et al., 2014). Another homolog, SNAP33, which forms a SNARE complex with SYP121 (Kwon et al., 2008), has been shown to be associated with response to mechanical stimulation and pathogen attack (Wick et al., 2003). Proteins involved in regulation of vesicle trafficking were also found in the PEF, e.g., two Rab GTPases and two SEC proteins (Supplementary Table S2). SEC6 and 15b are components of the exocyst complex in *Arabidopsis* (Hála et al., 2008) which tethers docking vesicles to the PM during exocytosis (Cvrčková et al., 2012). In tobacco, gene silencing of the exocyst subunits led to reduced callose deposition in response to a bacterial pathogen (Du et al., 2018). The *Arabidopsis* protein RabE has been shown to participate in the regulation of Golgi or post-Golgi trafficking of vesicles to the PM (Zheng et al., 2005). The PEF also contains a vacuole sorting receptor (VSR3), involved in vacuolar cargo sorting in *Arabidopsis* (Zouhar et al., 2010). A homolog to dynamin-related protein 1E (DRP1E) was also found in PEF (Supplementary Table S2). It likely functions in vesicle trafficking and has been shown to be important for polar cell expansion and cell plate formation (Kang et al., 2003).

Interestingly, DUF810 or PATROL1, was recently found to be involved in exocytosis and delivery of the cellulose synthase complexes to the PM by interaction with the exocyst machinery (Zhu et al., 2018). This protein was also found to be enriched in the PEF samples, which, however, did not show any enrichment in cellulose synthases (Supplementary Table S2). It is possible that PATROL1 is involved in more general delivery of carbohydrate synthase complexes at the cell surface and that it plays a similar role for callose synthase at the PD as it does for cellulose synthase at the PM.

A reticulone was also enriched in the PEF samples (Supplementary Table S2). This type of integral membrane proteins are involved in membrane curvature in the ER (Sparkes et al., 2010; Tolley et al., 2010) and were previously found to be located in the PD of *Arabidopsis* where it was suggested to form a highly curved membrane structure at the desmotubule (Knox et al., 2015).

Tetraspanins 1, 3, and 8 are another group of proteins that are enriched in PD (Fernandez-Calvino et al., 2011; Grison et al., 2015a). In animal cells they are scaffolding proteins involved in the formation of tetraspanin webs which are specialized domains in the PM (Charrin et al., 2009). Fernandez-Calvino et al. (2011) speculated that they may play a similar role in plants contributing to the organization of the PM at the PD. In mammals, tetraspanins have been shown to directly bind sterols (Charrin et al., 2003). This observation is consistent with the enrichment of the PD membrane in sterols and sphingolipids (Grison et al., 2015a).

Remorins are plant-specific scaffolding proteins enriched in plasma membrane DRMs (Mongrand et al., 2004; Raffaele et al., 2009; Srivastava et al., 2013). In keeping with our data, they have been found to be PD located (Raffaele et al., 2009) and affect PD conductivity when overexpressed (Raffaele et al., 2009; Gui et al., 2014; Perraki et al., 2014). A large group of proteins predicted to belong to the multiple C2 domain and transmembrane region protein (MCTP) family were enriched in the PEF (Supplementary Table S2). Members of this protein family have previously been localized to the PD and are most likely involved in macromolecular trafficking in plants (Fernandez-Calvino et al., 2011; Vaddepalli et al., 2014; Liu et al., 2018).

### Signal Transduction and Transporters

Different types of protein kinases were identified in the PD samples. Among the 28 enriched proteins, 20 were leucine-rich repeat protein kinases (Supplementary Table S2). Representatives from this group have been previously identified as PD located in rice (Jo et al., 2011). Two lysine motif (LysM) receptor kinases were also found in the PEF. These proteins function as cell surface receptors in chitin elicitor signaling, leading to innate immunity toward both biotic and abiotic stresses (Miya et al., 2007; Wan et al., 2008, 2012; Brotman et al., 2012). It is worth mentioning that no CDPKs were identified among the PD enriched protein kinases. Considering the important role of CDPKs in plant defense responses and development (Schulz et al., 2013) and the importance of Ca^2+^ in PD regulation, this class of proteins was expected to occur in the PEF samples.

A PDLP was also enriched in the PEF fraction. These membrane receptor-like proteins have been shown to regulate PD closure (Thomas et al., 2008; Lee et al., 2011; Carella et al., 2015). PDLPs also promote movement of viruses through PD when associated with viral movement proteins (Amari et al., 2010). Overexpression of *Arabidopsis* PDLP5 leads to PD closure whereas loss of its function increases permeability (Lee et al., 2011). It is also required for GSL4 mediated basal PD permeability and for GSL6 dependent responses to SA signaling at the PD (Cui and Lee, 2016).

Several transporters, including xanthine/uracil permeases, ATPases, sugar, metal, aquaporins and amino acid transporters, were significantly enriched in the PEF (Supplementary Table S2). A Ca^2+^-ATPase which contains a calmodulin binding autoinhibitory domain was also identified and it may be involved in maintaining calcium homeostasis through the transport of Ca^2+^ from the cytosol through the PM (Geisler et al., 2000).

## Conclusion

This study represents the first proteomic analysis of isolated PD structures from a tree species, revealing an enrichment of proteins specifically involved in responses to stress, transport, metabolism, and signal transcription. A new form of calcium-independent callose synthase activity was detected using biochemical assays, which suggests a possible control of PD function by calcium through the co-regulation of two types of callose synthase activities.

## Author Contributions

FL, VS, and VB conceived the study. FL, MM, QZ, and VS performed the experimental work and analyzed the data together with VB. FL, VS, and VB wrote the manuscript.

## Conflict of Interest Statement

The authors declare that the research was conducted in the absence of any commercial or financial relationships that could be construed as a potential conflict of interest.

## Footnotes

1 https://web.expasy.org/compute_pi/

2 http://www.arabidopsis.org/

3 http://suba.live/toolbox-app.html

## Abbreviations

APP: automated proteomics pipeline
BR: biological replicate
CDPKs: Ca^2+^-dependent protein kinases
CWs: cell walls
DRMs: detergent-resistant microdomains
DRP1E: dynamin-related protein 1E
ER: endoplasmic reticulum
ERD: early-responsive to dehydration
GH: glycoside hydrolase
GPI: glycosylphosphatidylinositol
GSL: glucan synthase-like
GTs: glycosyltransferases
LEAs: late embryogenesis abundant proteins
LRXs: leucine-rich repeat extensins
LysM: lysine motif
MF: microsomal fraction
MS: mass spectrometry
NPC: non-specific phospholipase
NSAF: normalized spectral abundance factor
PAPs: purple acid phosphatases
PD: plasmodesmata
PDLPs: plasmodesmata located proteins
PEF: plasmodesmata enriched fraction
PM: plasma membrane
PMEs: pectin methylesterases
RGP: reversibly glycosylated polypeptides
ROP: regulation of cell polarity protein
ROS: reactive oxygen species
SA: salicylic acid
SUSY: sucrose synthase
TEM: transmission electron microscopy
TMDs: transmembrane domains
UGT: UDP-glucose transferase
VSR3: vacuole sorting receptor.
