# Supplementary material for: Proteomic Analysis of Plasmodesmata From Populus Cell Suspension Cultures in Relation With Callose Biosynthesis
Source: Front Plant Sci. 2018 Nov 19;9:1681. doi: 10.3389/fpls.2018.01681 (PMC6252348; doi:10.3389/fpls.2018.01681)
Supplement: Supplementary file 1 [file Table_1.DOCX]

**Proteomic analysis of plasmodesmata from *Populus* cell suspension cultures in relation with callose biosynthesis**

Felicia Leijon, Michael Melzer, Qi Zhou, Vaibhav Srivastava and Vincent Bulone

**Supplementary Table S1:** Protocol for combined conventional and microwave-based fixation, dehydration and ultrastructural analysis.

| **1. Primary fixation** | 2.0% (v/v) glutaraldehyde and 2.0% (v/v) paraformaldehyde  in 0.05 M cacodylate buffer (pH 7.3); 12 h at RT | | | |
| --- | --- | --- | --- | --- |
| **Micowave processing in a PELCO Bio Wave®34700-230**  **(Ted Pella, Inc., Redding CA, USA)** | | | | |
| **Process** | **Reagent** | **Power**  **[W]** | **Time**  **[sec]** | **Vacuum**  **[mm Hg]** |
| **2. Wash** | 1x 0.05 M cacodylate buffer (pH 7.3)  and 2x distilled water | 150 | 45 | 0 |
| **3. Secondary fixation** | 1% (v/v) osmiumtetroxide in distilled water | 0  80  0  80 | 60  120  60  120 | 10  10  10  10 |
| **4. Wash** | 3x distilled water | 150 | 45 | 0 |
| **5. Dehydration** | Acetone: 30%, 40%, 50%, 60%,  70%, 80%, 90%,1x 100% | 150 | 45 | 0 |
|  | + 1 x Propylenoxide | + 5 min each on shaker | | |
| **6. Resin infiltration** | Spurr´s resin in propylenoxide:  25%, 50% and 75% | 2 h each | | |
|  | 100% Spurr | 12 h | | |
| **7. Polymerization** | 24 hrs at 70°C (oven) in Beam capsules | | | |
