# Supplementary material for: Proteomic Analysis of Plasmodesmata From Populus Cell Suspension Cultures in Relation With Callose Biosynthesis
Source: Front Plant Sci. 2018 Nov 19;9:1681. doi: 10.3389/fpls.2018.01681 (PMC6252348; doi:10.3389/fpls.2018.01681)
Supplement: Supplementary file 3 [file Data_Sheet_1.pdf]

Proteomic analysis of plasmodesmata from *Populus* cell suspension cultures in relation with callose biosynthesis

Felicia Leijon, Michael Melzer, Qi Zhou, Vaibhav Srivastava and Vincent Bulone

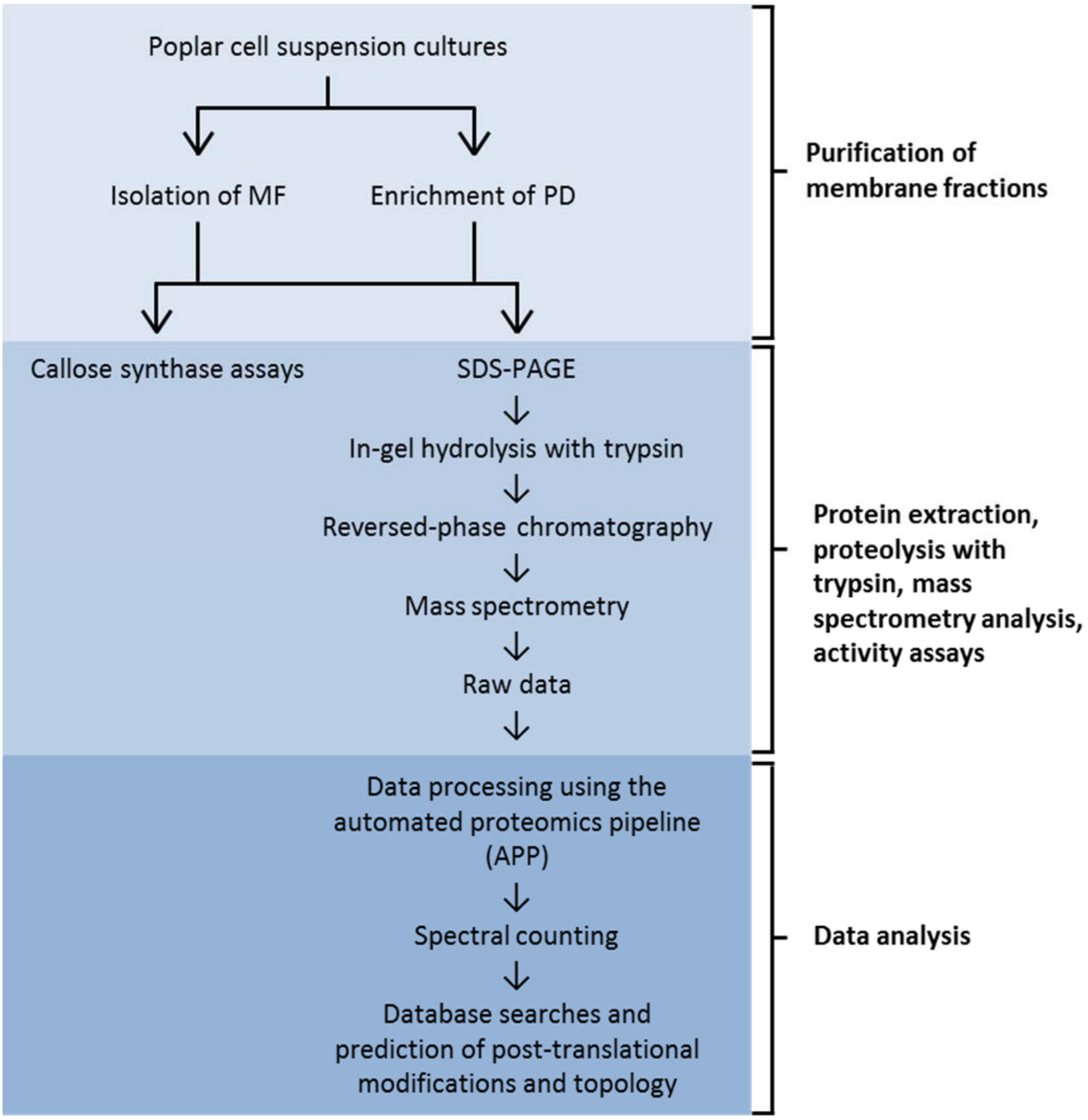

Figure S1. Experimental work-flow used for the purification of membrane fractions, protein extraction, proteolysis with trypsin, mass spectrometry and data analyses. For detailed description of data processing using the APP pipeline see Malm et al. (2014). MF, microsomal fraction; PD, plasmodesmata.

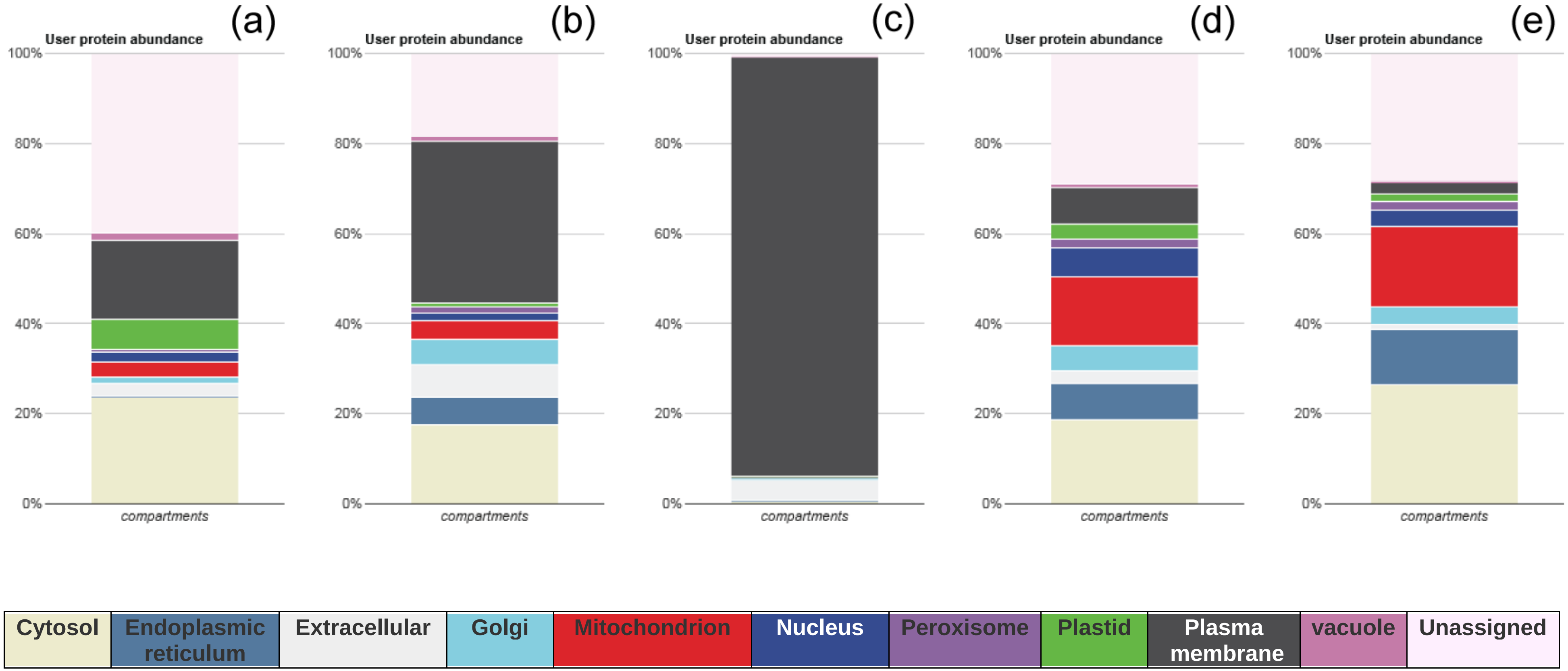

Figure S2. Estimation of the relative protein abundance of different cellular compartments using the Multiple Marker Abundance Profiling (MMAP) tool from the SUBA Toolbox using default settings (Hooper et al., 2017; http://suba.live/toolbox-app.html). Relative protein abundance for: all identified proteins from Fernandez-Calvino et al. (2011) (a); all proteins identified in the PEF sample (b); enriched proteins from the PEF sample (c); all proteins identified in the MF sample (d); and enriched proteins in the MF sample (e). The closest Arabidopsis homolog of each Populus gene was used for the estimation and only non-redundant proteins were used.

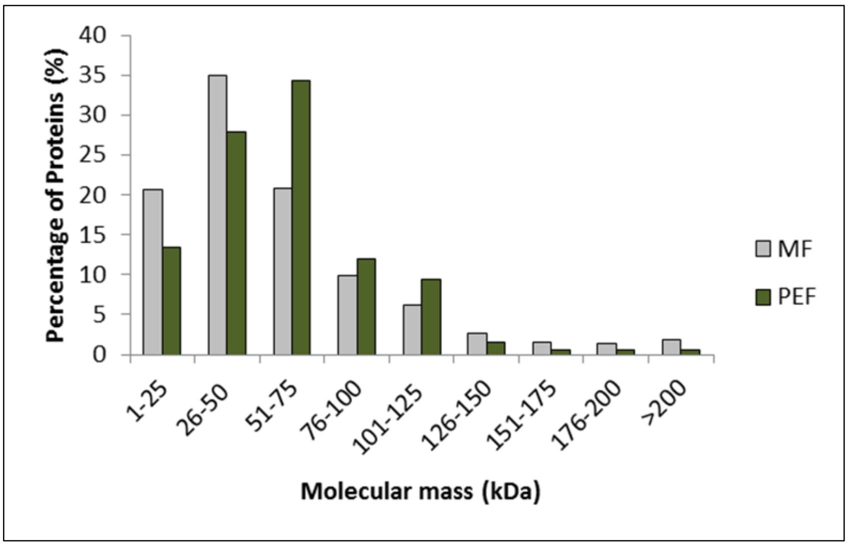

Figure S2a

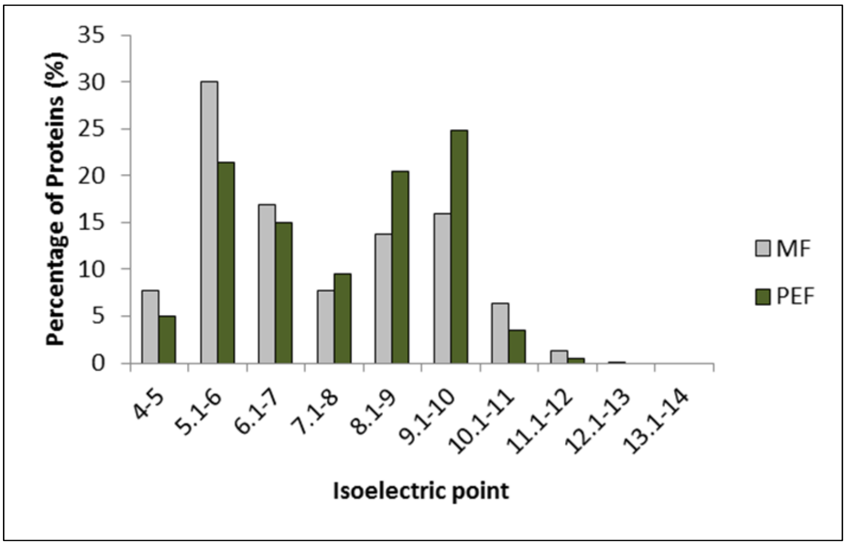

Figure S2b

Figure S3. Distribution of molecular mass (a) and isoelectric point (b) among proteins enriched in the microsomal fraction (MF) and the PD enriched fraction (PEF).

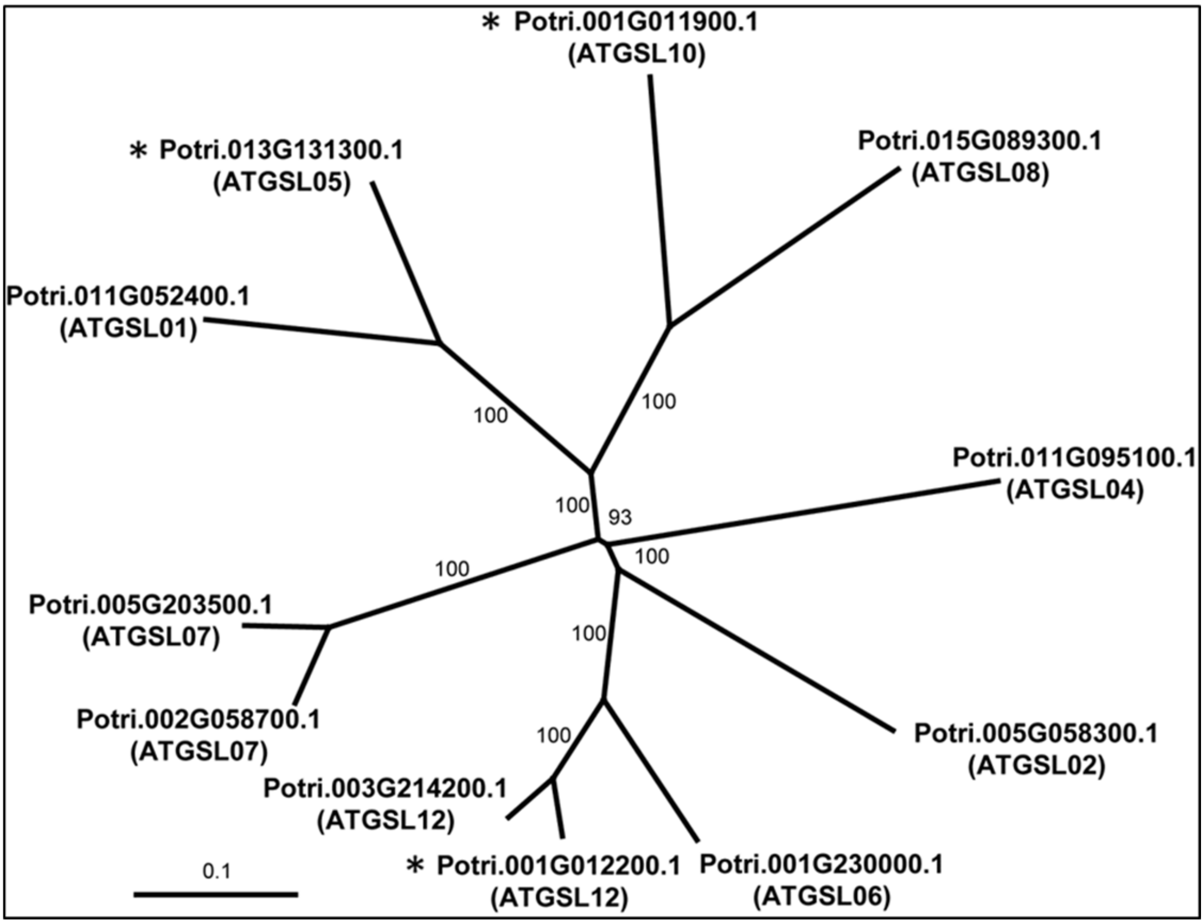

Figure S4. Phylogenetic tree of the putative glucan synthase like genes (*GSL*) in *Populus trichocarpa.* Accession numbers of all putative GSLs are shown. The names of the closest *Arabidopsis* homologs are given in brackets. AtGsl8/10 homolog (Potri.001G012000.1) present in both the MF and the PEF samples is not included in this tree as it was substantially shorter than the other GSLs genes with only two transmembrane domains. Proteins marked with asterisk were present in the PD enriched fraction. The phylogenetic tree was constructed using ClustalX version 2.1 (Larkin et al., 2007) with 100 bootstrap iterations. It was visualized with TreeView version 1.6.6 (http://taxonomy.zoology.gla.ac.uk/rod/treeview.html).

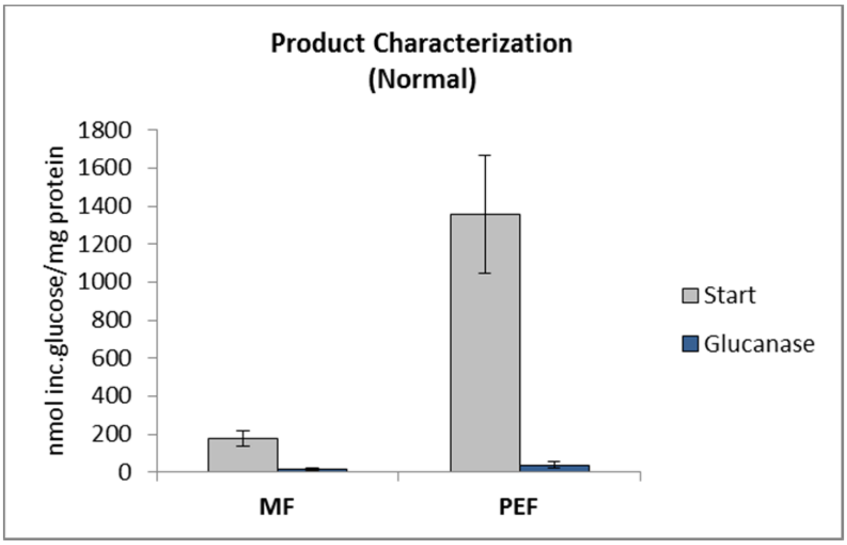

Figure S5a

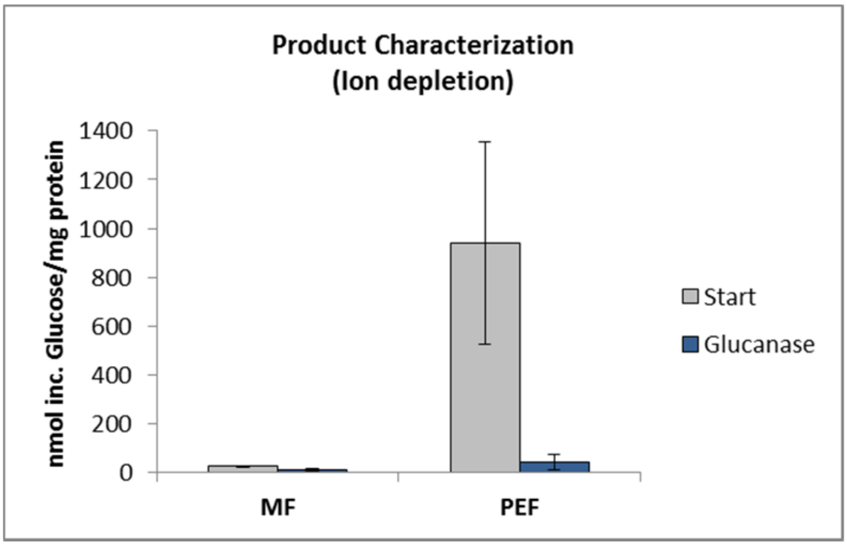

Figure S5b

Figure S5. Characterization of 1,3-β-glucan synthesized *in vitro* using enzymatic degradation with a specific endo-1,3-β-glucanase. Complete hydrolysis of the insoluble product after 5h of incubation with the glucanase confirms that the produced polysaccharide is a 1, 3-β-glucan under both Ca^{2+} dependent conditions (a) and ion depleted conditions (b). 'Start' samples correspond to amount of product present before addition of 1, 3-glucanase and 'Glucanase' samples show the amount remaining after hydrolysis. Assays including negative control samples which contain buffer instead of endo-1,3-β-glucanase were also performed (data not shown). MF, microsomal fraction; PEF, plasmodesmata enriched fraction. Data in Figures a and b arise from 3 biological replicates (BR) and 3 technical replicates from each BR. Bars indicate standard deviations.
